# Clinical characteristics, treatment, and wound management of pyoderma gangrenosum: A case series

**DOI:** 10.1371/journal.pone.0326203

**Published:** 2025-06-23

**Authors:** Feixia Wang, Lu Li, Weizhen Li, Xiaobo Ni, Zhe Pan, Lingling Ying, Mina Zhu

**Affiliations:** Nursing department, The First Affiliated Hospital, Zhejiang University School of Medicine, Hangzhou, China; Christian Medical College Vellore, INDIA

## Abstract

**Objective:**

This study aimed to evaluate the clinical characteristics, diagnostic processes, therapeutic approaches, and wound management strategies for patients diagnosed with pyoderma gangrenosum (PG), a rare inflammatory skin condition often resulting in misdiagnosis and treatment delays.

**Methods:**

A case series was conducted involving patients diagnosed with PG at the author’s Hospital, from January 1, 2020 to April 3, 2023. Data on patient demographics, wound characteristics, treatment methodologies, and wound care experiences were collected and analyzed.

**Results:**

The study included nine male patients with a mean age of 48.6 years (median 50 years). All patients were initially misdiagnosed, predominantly as skin infections or cellulitis. Lesions were mainly located on the lower extremities, with ulcerative PG being the most common subtype. Seven patients had skin injuries preceding PG onset. Treatment included systemic glucocorticoids and comprehensive wound care approaches. Three patients required additional interventions, like negative pressure wound therapy. Ultimately, six patients healed, while three died due to severe comorbidities or septic shock.

**Conclusion:**

PG is frequently misdiagnosed and often presents as ulcerative lesions triggered by skin injuries. Effective management, including systemic glucocorticoids and focused wound care, can significantly improve patient outcomes.

## Introduction

Pyoderma gangrenosum (PG) is a rare neutrophilic dermatosis characterized by inflammatory ulcers, commonly presenting as single or multiple rapidly evolving painful ulcers with purple margins and purulent drainage, which can lead to secondary infection [1]. The pathogenesis of PG is complex and related to neutrophil dysfunction, autoinflammatory responses, and abnormal immune function [2]. PG can affect individuals of any age, with a prevalence of 5.8 cases per 100,000 adults in the United States of America (USA) [3]. Delayed diagnosis and misdiagnosis are frequent, and PG is frequently misdiagnosed as necrotizing fasciitis, traumatic ulcers, vasculitis, or cutaneous cancers [4,5]. Inappropriate treatment can worsen the condition, leading to increased medical costs, prolonged hospital stays, and poor prognosis [6]. The high misdiagnosis rate is related to a poor understanding of the disease.

Mortality in patients with PG is high and may be related to comorbidities, severe secondary infections of chronic wounds, and immunosuppressive treatments [7,8]. The factors of poor prognosis include increased severity of disease, older age at diagnosis, unresponsiveness to treatment aimed at associated systemic disease, secondary infection, sepsis, the ulcerative (classic) variant of PG, and the bullous variant of PG, particularly if in a patient with hematologic abnormalities [9,10]. Nevertheless, PG remains a poorly understood disease because of its low incidence, and the exact factors associated with a poor prognosis remain uncertain.

Furthermore, the critical role of wound management in preventing secondary infection is often overlooked, and poor knowledge and experience in wound management of PG is key to poor patient outcomes [10,11]. Indeed, the initial management of PG should include optimizing wound care and pain management [12]. Topical therapy (potent or superpotent corticosteroids, tacrolimus, or cyclosporine) can be considered for mild and superficial lesions or as an adjunct to systemic medication for larger and deeper lesions, while systemic corticosteroids or systemic cyclosporine are frequently used as first-line treatment for large, multiple, or rapidly progressive PG lesions [1,10], but the evidence to guide management is limited, calling for additional data on patients with PG.

Therefore, this study aimed to review the clinical characteristics, diagnostic and therapeutic approaches, and wound management of patients with PG.

## Materials and methods

### Study design and patients

This case series study included patients with PG hospitalized at the author’s Hospital, between January1, 2020 and April 3, 2023. This study was conducted from May 3, 2023 to January 10, 2024. All patients were diagnosed based on the earlier diagnostic framework proposed by Su et al. [13], which focused on exclusion diagnosis: 1) the exclusion of skin lesions from other causes, 2) rapidly evolving painful ulcers, 3) with purple margins, 4) resolved significantly after hormonal therapy, and 5) pathological analysis revealing neutrophilic infiltration. Patients with a history of resolved PG prior to admission were excluded from the study.

This study was approved by the Ethics Committee of the First Affiliated Hospital, Zhejiang University School of Medicine (IIT20230284A). The requirement for informed consent was waived by the committee due to the retrospective nature of the study.

### Data collection and analysis

Information extracted from medical charts included sex, age, medical history, triggers, initial diagnosis, clinical variant of PG, ulcer location and characteristics, diagnostic process, laboratory test results, histopathological findings, treatments, and patient outcomes. The data were analyzed using descriptive statistics only.

## Results

### Demographics and clinical characteristics

The study included nine male patients with a mean age of 48.6 years (range: 23–71, median 50 years) (Table 1). The PG lesions were found on the limbs of seven patients (7/9), primarily in the lower extremities (5/7). Ulcerative PG was the most common (7/9) among the four subtypes (ulcerative, bullous, pustular, and vegetative). Seven patients developed PG following skin injuries, including venipuncture (2/7), debridement (3/7), and surgery (2/7). Two patients had underlying thrombocytopenia and acute myeloid leukemia (AML). Eight patients were admitted with systemic symptoms such as fever, chills, and fatigue.

**Table 1:** Demographics and clinical features of the patients.

#	Sex	Age	Causes	Previous history	Location	Skin Manifestation	Subtype	Pathology results	Laboratory tests
1	M	23	None	Carpal cartilage repair and ulnar shortening surgery	Face, limbs, and trunk	Red papuloid superficial ulcers without pain or itching	Vegetative	Unknown	WBC, CRP, and neutrophils% elevated ANCA negative
2	M	32	Debridement and NPWT for right lower extremity injury	None	Lower limb	Limb edema with elevated skin temperature. Well-demarcated violaceous round ulcer.	Bullous	Massive neutrophil infiltration Abscess formation	WBC, CRP, and neutrophils% elevated blood culture, PAS, GMS, and AFB stain negative
3	M	39	Debridement and NPWT after diagnosis of “cellulitis”	Respiratory failure	Lower limb	A rapidly progressive, violaceous, painful ulcer Partial tibia and stripped fascia were visible	Ulcerative	Massive inflammatory cell infiltration	CRP and neutrophils% elevated. Blood culture, wound secretion culture, ANCA, and GMS negative MRI excluded infection
4	M	48	Debridement, free skin graft, and NPWT after diagnosis of “shingles”	Tuberculosis	Lower limb	A rectangular lesion with scattered erosions at the edge Mild redness, little exudation, and tingling	Ulcerative	Superficial dermis edema Inflammatory cell infiltration (neutrophil predominant)	WBC decreased CRP and neutrophils% elevated Blood culture negative
5	M	50	Debridement after crusting of “cellulitis”	Thrombocytopenia Surgery of benign spinal tumor and cholecystectomy	Lower limb	A rapidly progressing ulcer with bloody and purulent secretions Marked redness, swelling, heat, pain, and purplish edge	Ulcerative	Diffuse neutrophilic infiltration	PLT decreased WBC, CRP, and neutrophils% elevated Wound secretion culture showed Klebsiella pneumoniae positive. MRI showed subcutaneous and muscle edema
6	M	54	None	Posthepatitic cirrhosis Hepatic failure Diabetes	Lower limb	Swollen and painful lower limb with partial skin ulceration and exfoliation Ruddy ulcer with purplish edge	Ulcerative	Unknown	WBC, CRP, and neutrophils% elevated ANCA, blood culture, and wound secretion culture negative MRI showed tissue edema
7	M	54	Venipuncture	Hypertension Acute myeloid leukemia	Upper limb	The venipuncture site was red, swollen, and painful, which rapidly enlarged to 11 × 10 cm with black scab and purulent secretions.	Ulcerative	Superficial dermis edema Scattered mononuclear cells and neutrophil infiltration	WBC elevated Neutrophils decreased% Blood culture negative
8	M	66	Venipuncture	Sepsis Hypertension IgG4-RD Sequelae of cerebral hemorrhage	Upper limb	The venipuncture site was red, swollen, and painful, which rapidly enlarged to 5 × 5 cm with dry black scab and purplish edge	Ulcerative	Unknown	CRP and neutrophils% were elevated Blood culture negative
9	M	71	Cholecystectomy	Pulmonary mycosis Splenectomy	Trunk	The laparoscopic incision site developed three well-demarcated, painful ulcers with moderate exudation and violaceous edge. Measuring 8.0 × 4.5 cm, 7.0 × 13.7, and 8.0 × 7.0 cm, respectively.	Ulcerative	Unknown	CRP elevated ANCA and blood culture negative

M: male; AFB: acid-fast bacteria stain; ANCA: anti-neutrophil cytoplasmic antibody; CRP: C-reactive protein; GMS: Gomori’s methenamine silver stain; IgG4-RD: IgG4 related disease; MRI: magnetic resonance imaging; PAS: periodic acid-schiff stain; PLT: platelet; NPWT: negative pressure wound therapy; WBC: white blood cell.

All nine patients were initially diagnosed with other skin conditions (Table 2), including skin infection (4/9), cellulitis (2/9), rash (1/9), and drug extravasation (2/9). The duration from symptom onset to the final definitive PG diagnosis varied from 9 to 42 days (median 18 days), including the time of misdiagnosis at a primary hospital. Eight patients were diagnosed by multidisciplinary collaboration, including the dermatology, infection, wound, and ostomy departments.

**Table 2:** Treatment and outcome of the patients.

#	Initial Diagnosis	Duration of diagnosis	Systemic therapy after PG diagnosis	Outcome
1	Rash	18 days	Methylprednisolone (40 mg/d, ivgtt) Oral methylprednisolone at discharge	Healing
2	Skin infection	25 days	Combined therapy: methylprednisolone (80 mg/d, ivgtt), IVIG (30g/d), adalimumab (80 mg/w, IH) oral methylprednisolone at discharge	Healing
3	Cellulitis	42 days	Combined therapy: methylprednisolone (80 mg/d, ivgtt), IVIG (30g/d), adalimumab (80 mg/w, IH) oral methylprednisolone+ methotrexate at discharge	Death
4	Skin infection	9 days	Methylprednisolone (40 mg/d, ivgtt) Oral methylprednisolone at discharge	Healing
5	Cellulitis	9 days	Combined therapy: methylprednisolone (80 mg/d, ivgtt), IVIG (30 g/d), adalimumab (80 mg/w, IH) Oral methylprednisolone+ cyclosporine at discharge	Healing
6	Skin infection	18 days	Methylprednisolone 40 mg/d (ivgtt) Treatment continued in a local hospital after discharge.	Death
7	Drug extravasation	19 days	Methylprednisolone 60 mg/d (ivgtt), reduced to 30 mg/d a week later Oral methylprednisolone at discharge	Healing
8	Drug extravasation	12 days	Methylprednisolone (40 mg/d, ivgtt)	Death
9	Skin infection	34 days	Combined therapy: methylprednisolone (60 mg/d, ivgtt), adalimumab treatment (80 mg/w, IH) Oral methylprednisolone at discharge	Healing

PG: pyoderma gangrenosum; IH: subcutaneous injection; ivgtt: intravenously guttae; IVIG: intravenous immunoglobulin.

### Laboratory findings

After admission, C-reactive protein (CRP) and whole blood cell count were performed in all patients. All nine patients had elevated CRP values, ranging from 12.90 to 237.01 mg/L (normal reference interval: 0–10 mg/L). On the other hand, white blood cell (WBC) counts showed heterogeneous results: they were elevated in five patients, normal in three, and decreased in one. The percentage of neutrophils increased in all but one patient with AML. Eight patients underwent blood culture and/or wound secretion culture. The wound secretion culture was positive for *Klebsiella pneumonia* in only one patient.

Biopsies were performed in five patients and showed predominantly neutrophilic inflammatory infiltration with local tissue edema and other nonspecific inflammatory changes.

### Systemic therapy

Glucocorticoid dosing was determined through multidimensional assessment incorporating ulcer dimensions, systemic comorbidities, and tissue invasiveness. For patients with corticosteroid tapering barriers or progressive ulcers following debridement, a combination of tumor necrosis factor-α (TNF-α) inhibitors and/or intravenous immunoglobulin (IVIG) was used as a steroid-sparing strategy. Four patients (4/9) with relatively mild conditions and limited ulceration were treated with a low dose of glucocorticoids (prednisone or methylprednisolone, 0.5–1.0 mg/kg/d) orally or intravenously. Five patients (5/9) with large lesions or acute progression were treated with methylprednisolone 1–1.5 mg/kg/d, which was switched to a low dose after 1–2 weeks. In addition, three received IVIG (30 g/d) and biologics (adalimumab, 80 mg/w) for immunomodulatory therapy, discontinued after symptom stabilization, and switched to single glucocorticoid therapy. After discharge, the patients were treated with oral glucocorticoids, in combination with immunosuppressants such as cyclosporine and methotrexate, when necessary.

### Wound management

For wounds with minimal exudation, innovative non-adherent lipid-colloid dressings were applied to prevent adhesion, minimize secondary injury, and alleviate pain. For wounds with significant exudation, hyper-absorbent hydrofiber dressings containing silver or alginate were used to manage drainage and reduce the frequency of dressing changes. Negative pressure wound therapy (NPWT), in conjunction with systemic glucocorticoid therapy, effectively controlled exudate and promoted wound healing. All patients reported varying levels of wound discomfort at rest and during dressing changes. To minimize irritation, wounds were irrigated with saline or diluted povidone iodine rather than wiped with cotton balls or gauze. For patients experiencing severe pain (visual analogue scale score of 4–7), lidocaine-based analgesics or tramadol were administered to provide relief.

### Patient outcomes

Six patients were healed, whereas the remaining three died of severe comorbidity (respiratory failure, hepatic failure, and sepsis) or secondary septic shock (Table 2).

#### Typical case #1.

**Fig 1:**
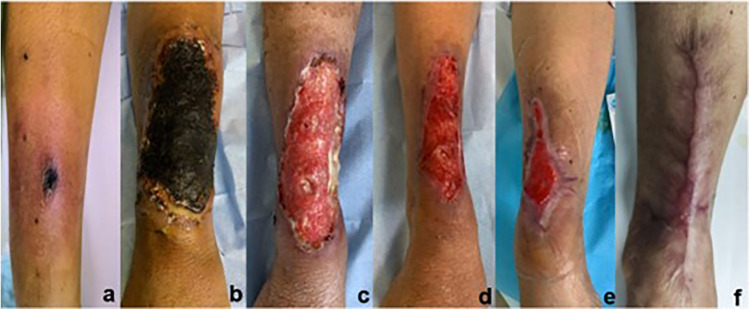
The wound of pyoderma gangrenosum on the arm of a patient with acute myeloid leukemia. (a. Redness, swelling, and black scab formation at the puncture site. b. The wound rapidly expanded to 13 × 9 cm. c. The black scab peeled off after 10 days of steroid treatment. d. Wound after 2 weeks of negative pressure wound therapy. e-f. The wound was shrinking until completely healed after 3 months).

A 54-year-old male with a history of hypertension was admitted for AML. The patient reported persistent tingling at the venipuncture site after an intravenous infusion in the right forearm. Three days later, the skin at the puncture site was red, swollen, and covered with a black scab (Fig 1a). A silver-based antimicrobial hydrogel and nano-silver medical antibacterial dressing were applied to promote autolytic debridement of the scab, while polymyxin and teicoplanin were used for systemic antibacterial treatment. The wound expanded to 13 × 9 cm (Fig 1b), and skin biopsy revealed mild hyperplasia, hypertrophy of the epidermis, superficial dermal edema, scattered mononuclear cells, and neutrophil infiltration. The patient was diagnosed with ulcerative PG after consulting with dermatologists and wound specialist nurses, and methylprednisolone was then administered intravenously at 60 mg/day (30 mg/Q12 hours). The black scab peeled off after 10 days of steroid treatment, revealing granulation tissue (Fig 1c) with massively purulent exudation. NPWT was performed by wound specialist nurses and was changed every 5–6 days, with negative pressure values of 80–100 mmHg. Two weeks later, the wound had shrunk to 9.0 × 6.5 cm (Fig 1d), and the exudation had significantly reduced; NPWT was discontinued. Silver-contained dressings, mesalt dressings (cleansing dressings with sodium chloride), or recombinant human epidermal growth factor gel was selected depending on the granulation tissue until the wound healed after 3 months (Fig 1e, f).

#### Typical case #2.

**Fig 2:**
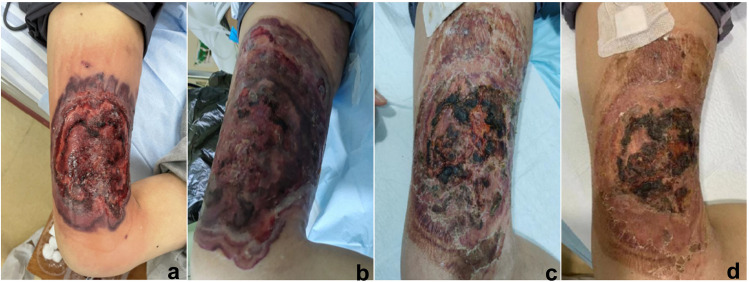
The wound healing process in the right thigh of a patient with pyoderma gangrenosum. (a. “Petal-like” ulcer measuring 20 × 15 cm on admission. b. Rapidly expanding wound despite systemic anti-infection treatment. c. Crusting of the wound edges 3 days after hormone treatment. d. Wound at discharge.).

A 50-year-old male patient was hospitalized with a history of cellulitis for over 1 month and a high fever for 6 days, with a maximum temperature of 39.9°C. The patient had been diagnosed with cellulitis at another hospital and received anti-infective treatment and debridement. Subsequently, the ulcer enlarged with massive exudation, and the patient was referred to the hospital. A “petal-like” ulcer of 20 × 15 cm was observed on the right thigh (Fig 2a). The ulcer was dull-red and violaceous with reddened surrounding skin, elevated skin temperature, and copious bloody and purulent exudation. Meropenem and vancomycin were used for systemic anti-infection treatment, and celecoxib for pain management. However, the ulcer remained uncontrolled (Fig 2b). The patient was diagnosed with PG following consultations with dermatologists, infectious disease specialists, rheumatologists, and wound specialist nurses, and methylprednisolone was added to the treatment. Three days later, the wound exudate was significantly reduced, and the edge was crusted (Fig 2c). The wound was exposed with a brace after flushing with normal saline and drying with gauze. The patient was discharged 1 week later in a stable condition (Fig 2d), and the ulcer completely healed 20 days after discharge.

## Discussion

The results highlight that PG is a rare and challenging condition often misdiagnosed as other skin disorders. Ulcerative PG was the most common subtype and triggers such as skin injuries were frequently observed. Effective wound management, including the careful handling of necrotic tissue, exudate, and infection, is essential to support wound healing in PG. Wound specialist nurses should deliver personalized care tailored to both the overall and local wound condition.

There is no clear consensus on the gender distribution of PG. Previous data from the National Inpatient Healthcare Databases in the USA and Spain have showed a male-to-female ratio of 1:1.40 ~ 1:2.02 [9,14]. However, the present study found a male predominance, consistent with the Korean study [7], which may be limited by regional and diagnostic criteria differences and sample size.

Consistent with previous studies [15,16], the current study also found that ulcerative PG was the most common subtype (7/9) and predominantly occurred in the limbs, especially the lower limbs. Skin injuries have been reported as a predisposing factor for PG. Here, seven patients (7/9) developed PG after skin injuries such as intravenous infusion, debridement, or surgery. Skin injuries can trigger the release of cytokines and danger signals, such as autoantigens and IL-36 and IL-8, which play a crucial role in the pathophysiology of PG [2]. The diverse clinical manifestations of PG in different stages and subtypes pose a challenge for clinical diagnosis. All patients in this study were initially diagnosed with other skin conditions such as infection, cellulitis, rash, or drug extravasation. Three patients who died had complications of respiratory failure, hepatic failure, and sepsis on admission, so the severity of comorbidities was considered to be the main cause of death in this group. These observations were consistent with the findings of Abdallah et al. [8], who found that 64% of patients with PG had comorbidities and that comorbidities were strongly associated with mortality. The diagnosis duration of patients with poor outcomes (3/9) ranged from 12 days to >1 month, and two of them had received debridement at other hospitals before definite diagnosis, which may worsen PG and increase the risk of death. Therefore, enhancing knowledge and early recognition of PG among medical professionals is essential to reducing delayed diagnosis and misdiagnosis.

Currently, diagnostic methods for PG are still being explored, and exclusion diagnosis is primarily used, including in the cases reported here. Maverakis et al. [17] developed and validated diagnostic criteria for ulcerative PG based on the Delphi method, with the primary diagnostic criterion being “neutrophil infiltration demonstrated by ulcer-edge biopsy,” in addition to eight secondary criteria. In 2019, Jockenhofer et al. [18] developed the PARACELSUS diagnostic tool, which considers rapid progression, assessment of relevant differential diagnoses, and reddish-violaceous wound borders as the major diagnostic criteria for PG. Four minor and three additional criteria were also identified, with a total score of ≥10 indicating a high probability of PG. Moreover, artificial intelligence (AI) technology has also been attempted to diagnose PG differentially. Birkner et al. [19] developed a deep convolutional neural network that showed a higher sensitivity for PG diagnosis than dermatologists after analyzing 422 wound photos of PG and leg ulcers. Nevertheless, more clinical studies are needed to validate the findings.

The systemic treatment of PG should be formulated based on several factors, including the severity of ulcers, related systemic diseases, patient status, and drug availability [20]. Glucocorticoids are considered first-line drugs for PG treatment because they can rapidly suppress inflammation by reducing the levels of proinflammatory cytokines, chemokines, and cell adhesion molecules [21]. A previous study showed that systemic glucocorticoid therapy at 0.5–1.0 mg/kg/d was effective in 40%−50% of patients with PG [22]. Nevertheless, the complete response rate is closely related to comorbidities and the severity of PG. Cyclosporine is another immunosuppressant commonly used for PG treatment and is reported to be as effective as glucocorticoids [1]. In addition to the above treatments, IVIG is often used as an adjuvant option in combination with glucocorticoids or immunosuppressants and plays multiple roles by regulating the innate immune system and enhancing the effects of glucocorticoids [23]. TNF-α is a pivotal pro-inflammatory cytokine that modulates chemokines including IL-1, IL-6, and IL-8, and promotes and maintains the inflammatory state through synergistic interactions [21]. Adalimumab and infliximab, commonly employed TNF-α inhibitors in PG treatment, can block the binding of TNF-α to its receptor and reduce levels of CRP, IL-1 and IL-6, thereby reducing inflammation. Zaman et al. [24] advocate the use of biologics including TNF-α inhibitors for severe or refractory PG cases, which is consistent with the systematic treatment principles in this study. Four patients with corticosteroid tapering barriers or ulcers progression post-debridement were treated with combination of TNF-α inhibitors and/or IVIG, and the remission rate was 75% (one patient died of severe respiratory failure).

The optimal treatment strategy for PG should include wound care, pain management, and systemic treatment. The “TIME” management principle, which focuses on tissue, infection, moisture balance, and epithelization, can be used to guide the wound care of PG, except for debridement [25,26]. Gentle and moderate cleaning to remove epithelial debris, fibrin, and necrotic tissue is necessary to promote wound healing [12]. The neutrophilic and inflammatory properties lead to excessive wound exudation, so hyper-absorbent dressings such as alginates, silver-contained dressings, or foams can be selected to control the exudation and avoid wound infection and edge maceration [27]. In this study, antimicrobial and hyper-absorbent dressings were the most common and could reduce the frequency of changes and painful irritation. NPWT can reduce edema, stimulate and promote the formation of wound granulation tissue and capillaries, and maintain appropriate wound humidity. Based on a literature review and treatment experience, excessive debridement or surgical treatment at the initial stage is not recommended, as it can stimulate inflammatory reactions and lead to worsened wounds [21]. Therefore, surgical debridement, skin graft, or NPWT should be performed at an appropriate time based on adequate glucocorticoids or immunosuppressive agents to avoid wound deterioration and maximize the effectiveness of surgical treatment.

### Limitations

Despite the rarity and low incidence of PG, the sample size in this study was limited to nine patients. The lack of a standardized diagnostic tool for PG in China necessitates reliance on exclusionary diagnosis, which remains the primary approach in this study. Additionally, the retrospective nature of the study further introduced heterogeneity within the patient population, encompassing all individuals diagnosed with PG. To address these limitations, future prospective studies are needed to better define the optimal timing for employing NPWT and surgical interventions in PG management.

## Conclusions

PG is a rare condition characterized by heterogeneous clinical features and non-specific indicators, making it highly susceptible to misdiagnosis. This can lead to adverse outcomes, such as wound deterioration or even amputation, due to inappropriate treatment. Accurate diagnosis and effective management of PG require multidisciplinary collaboration, with early recognition being crucial to preventing misdiagnosis and its associated complications. Alongside systemic therapy, meticulous and gentle wound care is essential. This includes the personalized use of advanced medical dressings and addressing pain management to enhance patient compliance and comfort. In the future, it is necessary to systematically assess the differences in efficacy and safety between traditional drugs (single/combination regimens) and targeted therapies such as biologics by large-sample randomized controlled trials, as well as to construct standardized integration strategies of systemic therapy and local interventions (e.g., antimicrobial dressings, NPWT) under the multidisciplinary mode.
